# A guide to acquired vitamin K coagulophathy diagnosis and treatment: the Russian perspective

**DOI:** 10.1186/s40199-017-0175-z

**Published:** 2017-04-17

**Authors:** Valery V. Wojciechowski, Daniela Calina, Konstantinos Tsarouhas, Alexander V. Pivnik, Alexander A. Sergievich, Vladimir V. Kodintsev, Ekaterina A. Filatova, Eren Ozcagli, Anca Oana Docea, Andreea Letitia Arsene, Eliza Gofita, Christina Tsitsimpikou, Aristidis M. Tsatsakis, Kirill S. Golokhvast

**Affiliations:** 1 Department of Hospital Therapy and Pharmacology, Amur State Medical Academy, 675000 Blagoveshchensk, Russia; 2 Department of Clinical Pharmacy, University of Medicine and Pharmacy of Craiova, Petru Rares, 200349 Craiova, Romania; 3 Cardiological Department, University Hospital of Larissa, Larissa, Greece; 4 Department of Hematology-Oncology and Secondary Immunodeficient Diseases, D.D. Pletnev Moscow Clinical Research and Practical Centre of Health Department, 111123 Moscow, Russia; 5 School of Arts, Culture and Sports, Far Eastern Federal University, 690022 Vladivostok, Russia; 6 School of Biomedicine, Far Eastern Federal University, 690022 Vladivostok, Russia; 7 Department of Hematology, Amur Regional Clinical Hospital, 675000 Blagoveshchensk, Russia; 8 Department of Pharmaceutical Toxicology, Faculty of Pharmacy, Istanbul University, Beyazit, Istanbul, 34116 Turkey; 9 Department of Toxicology, University of Medicine and Pharmacy of Craiova, Petru Rares, 200349 Craiova, Romania; 10 Department of Pharmaceutical Microbiology, Faculty of Pharmacy, “Carol Davila” University of Medicine and Pharmacy, 6, TraianVuia Street, sector 2, 020956 Bucharest, Romania; 11 Department of Hazardous Substances, General Chemical State Laboratory of Greece, Mixtures & Articles, 16 An. Tsocha Str, Athens, 115121 Greece; 12 SEC Nanotechnology, Far Eastern Federal University, 690022 Vladivostok, Russia; 13 Forensic Sciences and Toxicology Department, Medical School, University of Crete, P.O. Box 1393, 71003 Heraklion, Crete Greece; 14 Scientific Educational Center of Nanotechnology, Federal Scientific Center of Hygiene, Far Eastern Federal University, F.F. Erisman, Moscow, 690950 Russian Federation

**Keywords:** Vitamin K, Acquired coagulopathy, Rodenticides

## Abstract

**Abstract:**

Physicians often come across with cases of vitamin K antagonists–dependent coagulopathy for reasons such as accidental use of the vitamin K antagonists (VKA), excessive administration of prescribed anticoagulants of indirect action or not reported administration of vitamin K antagonists due to memory impairment and/or other mental disorders, even deliberate use thereof (attempt to murder or suicide). Rodenticide-poisoning (coumarins, warfarins) via food or occupational accidents are difficult to diagnose. This article discusses different types of acquired vitamin K-dependent coagulopathy. Differential diagnosis is primarily based on patient statements before additional causes of vitamin K deficiency are explored. Even when pathological vitamin K deficiency is not determined, appropriate and urgent medical treatment is necessary: administration of fresh frozen plasma or concentrated factors of the prothrombin complex, administration of vitamin K remedies along with symptomatic therapy. With early diagnosis and prescription of appropriate therapy, prognosis is favorable.

**Graphical abstract:**

Reasons for vitamin K antagonists–dependent coagulopathy cases
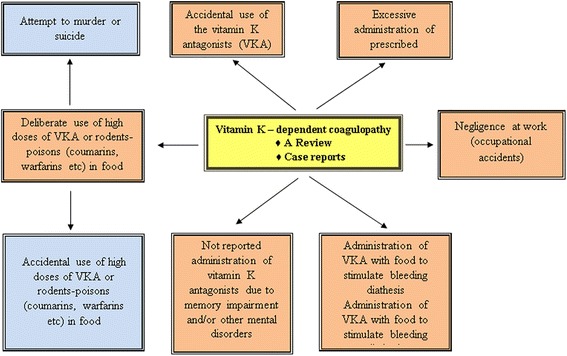

## Background

Vitamin K-dependent coagulopathies are rather common in clinical practice, especially in cases of overdose of orally prescribed anticoagulants of indirect-action, also known as the vitamin K antagonists (VKA). Vitamin K deficiency can be due to deficient biosynthesis in the intestinal tract that leads to deficient bioavailability in various pathological situations such as: medicine induced intestinal dysbacteriosis, especially after antibiotic treatment; enteropathies accompanied by heavy diarrhea; mechanical jaundice with acholia that leads to reduction or absence of bile secretion in the intestinal tract; prematurity of the newborns associated with subnormal synthesis of vitamin K in intestinal tract; severe liver disease (acute dystrophies, hepatitis, cirrhosis); autoimmune disorders due to double antiviral therapy with Peginterferon and Ribavirin in patients with hepatitis C virus infection [1].

Physicians occasionally come across with cases of vitamin K-dependent coagulopathy of quite different origin: not reported use of VKA by patients with psychiatric disorders; accidental use of indirect anticoagulants instead of other prescribed medication; in cases of senile amnesia excessive use of VKA; intoxications with rodenticide-poisons containing VKA; illegal cases of VKA usage with the purpose of murder or suicide. Even though such cases are not frequent, physicians can describe similar reports from their personal experience.

The aim of this paper is to perform a thorough literature review concerning all types of artificial vitamin K-dependent coagulopathies and to present representative clinical cases from medical practice.

## Vitamin of coagulation – vitamin K

The first observations on K vitamins action, namely massive bleeding into the subcutaneous tissue, muscles and other organs, were observed in the 1920’s and 1930’s in animals (chickens and birds) fed with cholesterol/fat-deficient food. Subsequent feeding with plant products developed a curative effect. During the same period, an outbreak of a cows’ diseases (“disease of sweet clover”) in the northern part of the USA and Canada was found to be associated with consumption of moldy silage from *Melilotus* (sweet clover) by the animals with clinical signs identical to the previously described hemorrhagic diathesis of chickens [2, 3]. In 1939, a group of researchers, under the supervision of the Swiss scientist Karrer, for the first time separated vitamin K1 from the plant alfalfa (*Medicago sativa*), under the chemical name of phylloquinone, which is a fat soluble polycyclic aromatic ketone, stable to air and moisture but decomposes in sunlight. The same year, the American biochemists Binkley and Doisy managed to isolate from the spoilt fish-flour the vitamin K2 or menaquinone with similar to vitamin K1 anti-hemorrhagic properties, but with different absorption profile and a more complex range of activity. Apart from the natural K-Vitamins (K1 and K2), there are a number of naphthoquinone derivatives, which are acquired synthetically and possess similar anti-coagulative effect (Vitamins K_3_–K_7_).

It wasn’t before 1939 that the crystals of dicumarol, a natural chemical substance of combined plant and fungal origin, were isolated. Dicumarol is a derivative of coumarin, a bitter-tasting but sweet-smelling substance made by plants that does not itself affect coagulation, but when transformed in mouldy feeds or silages by a number of species of fungi, into active dicoumarol, which does affect coagulation, and was discovered in mouldy wet sweet-clover hay, as the cause of a naturally occurring bleeding disease in cattle.

Vitamin K belongs to lipophilic and hydrophobical vitamins group. Vitamin K participates in the carboxylation of glutamic acid residues in polypeptide chains of certain proteins to form gamma-carboxy glutamic acid (Gla-radicals). Gla-radicals, being part of the Gla-proteins, play a major role in several biological activities, such as blood clotting, bone metabolism, connective tissue formation and kidney functioning. The said vitamin participates in the absorption of calcium due to the two free carboxyl groups of the Gla-radicals and facilitates interaction of calcium with Vitamin D. There are protein structures in the heart and lungs, which can be synthesized only with the involvement of vitamin K.

**Fig. 1:**
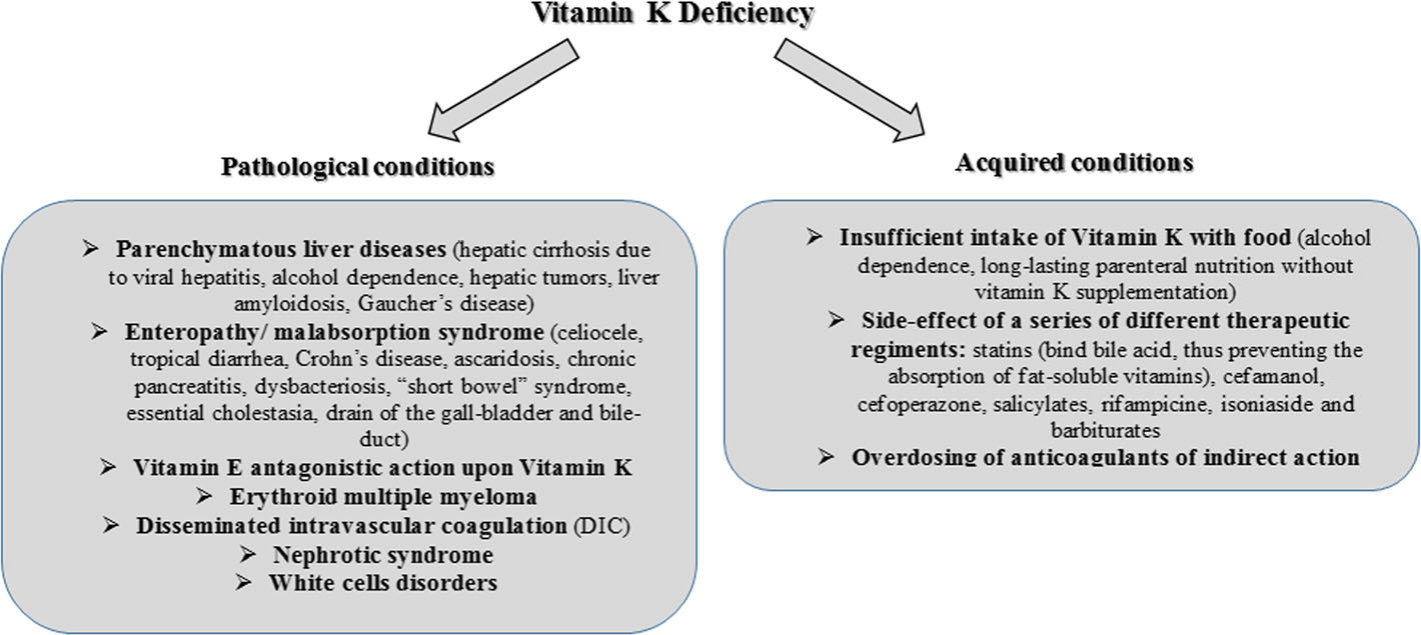
Vitamin K deficiency

Reasons for Vitamin K-deficiency in humans can vary (Fig. 1). In current clinical practice it is very useful to determine the cause of coagulopathy before appropriate treatment is administered.

### Overdosing of anticoagulants of indirect action

Several derivatives of coumarin, such as 4-hydroxy coumarin, have similar anticoagulant properties. The first discovered drug from the class of indirect anticoagulants was dicumarol and it was patented in 1941. Coumarin-like anticoagulants have also been used as poisons for gnawing animals. Warfarin, synthesized and registered as poison for gnawing animals in the USA in 1948 rapidly gained widespread acceptance [2]. Soon enough, at 1951, warfarin was used in a suicide attempt by a USA army conscript who, in hospital, was administered with Vitamin K as a specific antidote [2]. With time rodents have developed resistance to warfarin and several long-acting coumarin derivatives (the so-called superwarfarin anticoagulants, such as brodifacoum, diphenadione, chlorophacinone, and bromadiolone) were developed. At the same time warfarin as indirect anticoagulant has become widely used in the clinical practice.

**Fig. 2:**
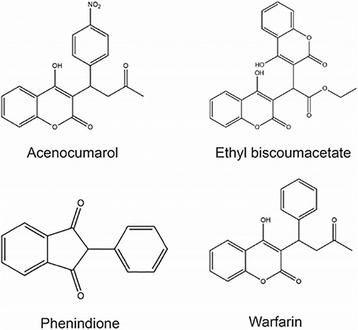
Warfarin and acenocoumarol mechanism of action

At a molecular level, when a patient receives warfarin, levels of prothrombin and the liver-formed VII, IX and X factors in plasma start decreasing, indicating that warfarin suppresses the hepatic synthesis of these molecules. At the same time, synthesis of the two physiological anticoagulants – C and S proteins – is blocked. Warfarin competes with Vitamin K, thus blocking Vitamin K action. Vitamin K is necessary for the carboxylation at the final stage of synthesis of the above mentioned clotting factors. Through the carboxylic group the clotting factors bind to calcium, and through calcium with phospholipids and factor Xa. The latter is necessary for the transformation of prothrombin into thrombin. At the beginning of warfarin administration, the process of clotting is not blocked immediately, as there is a “stock” of circulating prothrombin and relevant clotting factors. Drug’s maximum action appears on the 3^rd^ – 5^th^ day from the beginning of its administration and ends 3–5 days after cessation. Similarly, symptoms of K-hypovitaminosis develop successively, depending on the half-life of the clotting factors involved, starting from the decrease in activity of VII factor (half-life 4 h), followed by factors IX and X activity, and finally prothrombin activity decreases in 3–4 days. In the same order the restoration of procoagulants levels is achieved after the compensation of Vitamin K-deficiency (Fig. 2).

**Fig. 3:**
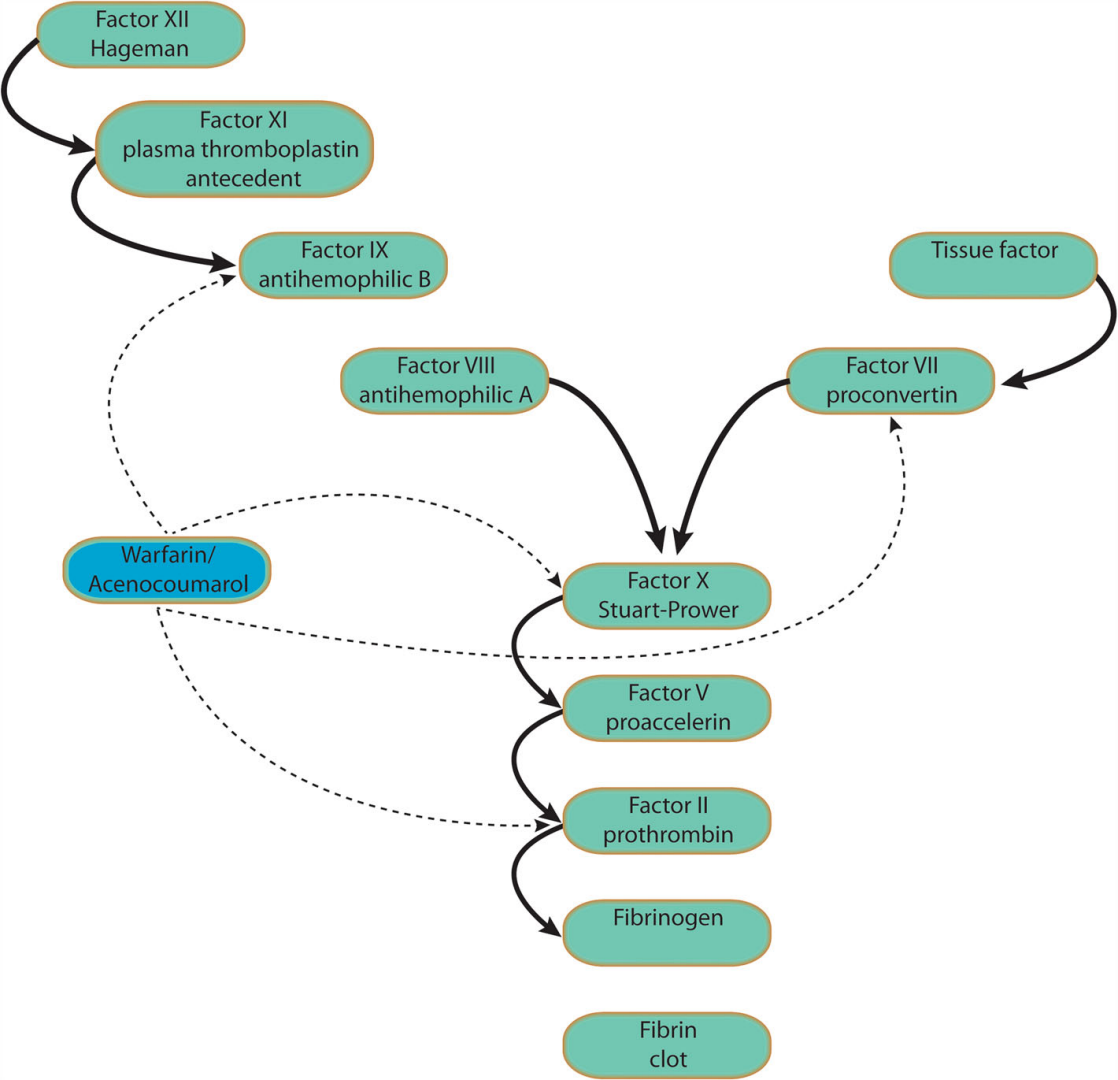
Chemical structures of anticoagulants of indirect action

According to their chemical structure, anticoagulants of indirect action are classified into three groups (Fig. 3):

- ✓derivatives of monocoumarine (warfarin, acenocoumarol);
- ✓derivatives coumarin (ethyl biscoumacetate);
- ✓derivatives of indandione (phenindione)

The indandione derivatives possess anticoagulant action like coumarins but more often cause side effects such as liver toxic effects and skin manifestations. This is why the indandione derivatives usually are prescribed to patients with allergic reactions to coumarin derivatives. Alternatives to warfarin are oral anticoagulants, such as direct thrombin inhibitor dabigatran [4] and direct inhibitor of the Factor Xa – rivaroxaban [5] and apixaban [6].

The initial dose of warfarin is 5 mg/day, and the administration schedule is further individualized depending on the prothrombin time and/or international normalized ratio (INR). Genotype determination of CYP2C9 and VKORC1 could facilitate regulation of warfarin dosage [7]. In general, warfarin dose should be reduced or even ceased, INR monitoring should become more frequent and the patients should be examined for the presence of bleeding symptoms and erythrocytes in urine. Hemorrhagic risk rises steadily with INR increase: at values < 2.5 hemorrhages are very rare and from 2.5 to 3.0 are rare and usually minimal. Prothrombin time during warfarin administration should be increased 2–4 times compared to initial values and INR in most cases should range between 2 and 3.

**Fig. 4:**
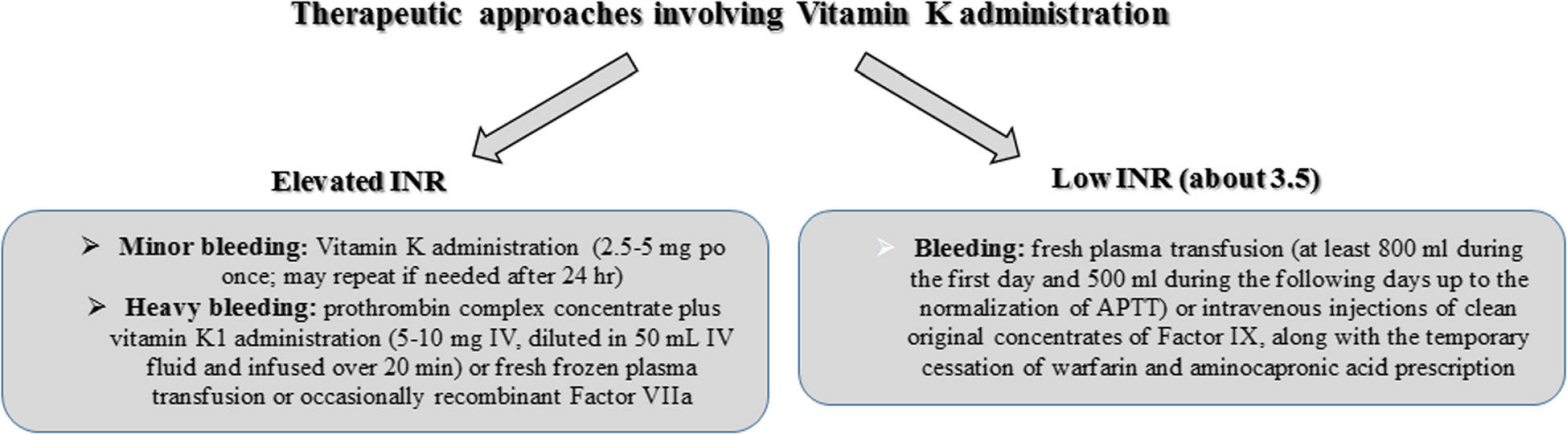
Therapeutic approaches involving Vitamin K administration

Several approaches are followed in cases of elevated INR depending on INR values and the hemorrhagic diathesis of the patient. Usually low INR implies either Factor IX abnormality (as determined by activated partial thromboplastin time – APTT), sensitivity to clotting factor deficiencies, or conditions predisposing to bleedings (e.g. peptic ulcer disease, erosive gastritis etc) [8–12] (Fig. 4).

During the first days of warfarin intake, massive thrombosis may develop accompanied by skin necrosis and by limbs gangrene. In this case protein C plays a decisive role, as it is the first to deplete, thus potentially lead to a paradoxical increase of prohtrombotic diathesis. In order to avoid this phenomenon, co-administration of warfarin with heparin is recommended at the beginning of the treatment [13, 14].

The overdosage of VKA or the so-called “artificial coumarinic bleeding” is often presented in clinical practice and it is easy to handle analyzing the appropriate laboratory tests and patient’s statements which are considered crucial [15–17].

Apart from human medicines, warfarins have found application in domestic hygiene, too. A number of different chemicals are used as rodenticides (RDs, group of zoocids), such as aluminium and zinc phosphide, arsenic, thallium, strychnine, barium carbonicum, along with indirect anticoagulation agents [18]. The most widely used RDs are the 1,3-indandiones and the 4-hydroxy coumarin classes. They act selectively as anticoagulants.

The majority of modern RDs with chronic action are indirect anticoagulants – Vitamin K antagonists. As a general rule they are water insoluble, but they also dissolve in organic solvents; they are not biodegradable and they possess interesting cumulative properties as they are concentrated in small quantities in animals’ tissues until lethal doses are achieved. The mechanism of action of the majority of RD is the inhibition of Vitamin K which participates in the synthesis of blood-coagulation factors. Typically the beginning of clinical symptoms varies from 3 to 5 days from the first RD exposure due to the natural reserves of Vitamin K necessary for the synthesis of blood-clotting factors. The gradual cessation of Vitamin K synthesis leads to the development of a severe hemorrhagic syndrome, accounting for animals’ death. The concentration of the active substance (AS) in baits is so minimal, that no defensive reaction occurs and large amounts of baits are usually applied [19]. The baits are pre-prepared with neutral fillers painted in different colors to recognize them visually. In addition, to prevent the accidental poisoning of people and pets, bitterness (Bitrex) is added. The ASs of these rodenticides and the concentrated commercial preparations with high dermal absorption are classified to higher tier hazard classes as carcinogens, reproductive toxicants and mutagens [19]. The other commercially available preparations are classified to less severe hazard classes for their dermal effects (irritation and sensitization) and their irritant effects upon the mucous membranes of the eyes.

The first-generation anticoagulants (warfarin RD) work slowly (death of gnawing animals delay for a month) and require a range of repeated applications (from 3 to 6 times). De-ratization time delays 15–20 days, sometimes a month.

The second-generation anticoagulants - superwarfarins (brodifacoum, bromadiolone, flocumafen, difetialon and difenacoum) work quicker: the animals’ death occurs in 4–10 days with a single consumption of the bait. Superwarfarins were developed to control gnawing animals resistant to warfarin action. Unlike warfarin, which is easily removed from the body, superwarfarins accumulate in the liver and kidneys after their consumption.

The contact of workers and citizens with the active substances and commercial forms of RDs could occur in different stages of preparation and use, taking into account their cumulative action and their long half lifetime in the environment of approximately 157 days. Usually in cases of intoxication described in the literature, contact with RD agents took place after their use at home, at work or by direct contact with RDs contained in other substances [20–23]. In isolated cases, poisoning was a part of suicidal attempts [24]. In addition, cases of poisoning with superwarfarins in patients with mental disorders under suicidal crisis are often described [25].

Poisonings are generally classified as premeditated, accidental and of unknown etiology [26]. RD human poisoning is rare in comparison with other toxic substances, but in continue rise [27–29]. In 1988 in the USA, 5133 cases of superwarfarin poisonings were registered due to accidental administration, suicidal attempts and psychiatric disorders symptoms (Munchausen Syndrome), while in 1995 cases raised to 13423 [30]. In addition, in 2004 more than 16000 cases of RD poisoning, among them 15000 in children, were recorded in the USA [26]. In 2015, data on rodenticides containing indirect coagulants poisonings in the USA over a period of 25 years were published. 315951 cases were examined, from which nearly 90% of the subjects were children in accordance with other published data [31]. Different routes of accidental administration were identified: oral, inhalation, intradermal and others. Only in 2% of all cases hemorrhagic syndrome and a fatal outcome was observed [32].

In another interesting study, 31 case reports of superwarfarins poisoning (19 men and 12 women), with average age 48 years (range from 2 to 88 years) were reported: brodifacoum (*n* = 21), flocumafen (*n* = 5), bromodiolone (*n* = 2), coumatetralyl (*n* = 1) were involved while 2 cases were of unknown origin. Thirteen patients had psychiatric disorders, including depressive disorders (*n* = 5), dementia (*n* = 5) and cognitive deterioration (*n* = 1). According to the patients’ statements, eleven patients (35.5%) took medicatons which can potentiate the Vitamin K antagonist, including counter-depressants (*n* = 6); non-steroid anti-inflammatory drugs (*n* = 3); disaggregants (*n* = 3) and antacids (cimetidine, ranitidine, *n* = 2). Two patients abused herbal therapy but no one has been diagnosed with hepatic disorders. Ten patients accidentally suffered from rodenticide impact, while 21 patients used rodenticides in suicidal attempts and 11 patients were poisoned by rodenticides in a state of alcoholic intoxication (35.5%). Being in an alcoholic condition the moment of intoxication intensifies coagulopathy and influences the genetic susceptibility to warfarin [33]. Among the 31 subjects only one patient presented hemorrhagic symptoms (hematomas and hematuria). Nevertheless, 11 of 31 patients (35.5%) were diagnosed with laboratory disorders typical of VKA poisoning [34].

Criminal cases involving K-dependent coagulopathies can be found in the literature since the 1970-80s, where VKA used as medicines or rodenticides containing VKA were used with the purpose of murder or suicide [35–37].

In a review of cases and suicidal attempts using rodenticides in Yugoslavia from 1968 to 2000, 88 cases were described using Zn phosphide or rodenticides containing VKA [38]. Pupils of the primary school, people using alcohol excessively and patients with mental depressions, neurosis and other mental disoders were identified. Women were more susceptible than men to suicide attempts using rodenticides.

Even a single dose of superwarfarins, that are 100 times stronger than warfarin, can cause signs of poisoning with severe consequences in time; even 1 mg of superwarfarin can lead to coagulopathy through/by Vitamin K-deficiency [39]. Superwarfarins could cause long-lasting coagulopathy from several weeks till several months [39, 40].

Accidental RD poisoning is characterized by hemorrhagic syndrome (hemorrhages on the skin, nasal hemorrhages, bloody stools, hematuria, blood spitting), weakness, paleness, breathlessness, anorexia, vomiting, syncope, abdominal pain. Hematuria is one of the most common clinical signs of superwarfarins’ poisoning [39]. Some researchers also describe the presence of hemorrhagic coagulopathy associated with the paradoxical blood-clotting in the left fossa poplitea area (obviously associated with deficiency of the natural anticoagulant protein C) [34, 41].

The diagnosis of superwarfarin-induced coagulopathy is difficult because the toxic agent is hard to identify and the early clinical signs are not always specific [23]. The diagnosis is based on the patient’s statements, the clinical signs and blood-tests for anaemia, thrombocytopenia, hypoproteinemia, elevation of the alkaline phosphatase and moderate elevation of liver enzymes. In addition, coagulogram could reveal an increase in INR, prothrombin time and activated partial thromboplastin time (A-PPT) and decrease of II, VII, IX and X blood-coagulation factors levels.

When the patient with coagulopathy of unknown etiology appears at emergency departments, differential diagnosis is necessary among warfarin poisoning, disseminated intravascular coagulation (DIC) syndrome, severe celiocele accompanied by mal-absorption, vitamin K-deficiency or contact with pathological inhibitors of coagulation [23, 42]. In the differential diagnosis of coagulopathy with a background of liver abnormality, it’s necessary to take into account the fact that liver disease may depress not only the K-dependent factors but also the Vitamin K-independent factor V, an observation which simplifies the differentiation between hepatic and non-hepatic forms of the hemorrhagic syndrome [43].

The presence of superwarfarin can be proven with the use of liquid chromatography [44, 45], but unfortunately many health care centers do not have access to these technologies and this is why administration of high doses of Vitamin K inhibitors in case of suspicion of RD poisoning is recommended [26]. In the literature in the majority of poisoning cases no sign of residual pathology appears, even in severe cases. Death and other serious complication are rare [28, 31]. Only 8 (0.08%) fatal cases were registered among 79025 cases of superwarfarins poisonings reported on a period of 8 years. In a published review of 24 cases of brodifacoum poisoning, 6 (25%) individuals died after acute intoxication [46].

Instructions to address superwarfarins poisonings are generally provided by the National Poisoning Centre and may include, depending on the nature and hazard classification of the AS and the preparation, hydration and/or administration of a solution of potassium permanganate (1:5000, 1:10000), induce vomiting, administration of activated carbon and saline purgative (20–25 g of sodium sulfate in a glass of water), administration of Vitamins K_1_ or K_3_ as antidotes, washing off the skin and eyes abundantly with water or 2% solution of sodium bicarbonate and administration of 1–2 eye drops of 30% solution of sulfacyl sodium (albucid). The importance of poisonous-agent-specific treatment has also been highlighted in the literature [38]. Zn phosphide poisoning demands stomach lavage with a solution of sodium bicarbonate and administration of activated carbon, laxatives or diuretics. In cases of VKA poisonings treatment of K-dependent coagulopathy is recommended. Of high importance for a physician when diagnosing a coagulopathy induced by superwarfarins is to exclude its intentional administration either for suicide or for murder [47]. As the half-life of warfarin is 17 h, administration of Vitamin K-preparations (prothrombin complex concentrate) and/or transfusions with fresh frozen plasma allow to correct INR. On the other hand, in cases of poisonings by superwarfarins with a half- life of about 69 days, after stopping the therapy with Vitamin K INR rises again.

In the majority of the cases, patients with Munchausen Syndrome physically harm themselves to simulate hemorrhage [48]. There are documented cases when these patients take both oral Vitamin K antagonists and rodents-poisons containing Vitamin K antagonists [36, 49, 50] in order to simulate the hemorrhagic syndrome.

Munchausen Syndrome by Proxy (MSBP) is a type of factitious disorder when parents or people having another person’s custody, usually women, intentionally induce or imagine a disease state for a child or a vulnerable adult, in order to request medical assistance [51–53]. The most common clinical manifestations linked to MSBP are bleedings, diarrhea, vomiting, poisonings, infections, breathlessness, fever, allergies and the Sudden Infant Death Syndrome [54]. Cases of K-dependent children coagulopathies induced by relatives are also reported [55].

As it’s practically impossible to analyze patient history from patients with Munchausen Syndrome, the proper treatment should be immediate diagnosis with K-dependent coagulopathy without waiting for the blood results of the rodenticide definition [56].

A series of clinical cases are reported.

## Clinical case reports

**Table:** 

*Case report 1*
*A 60-year-old male patient, blind, was transferred to hospital with a diagnosis of mediastinal mass found during chest X-ray.* *During examination all clinical and laboratory signs of Vitamin K – dependent coagulopathy were diagnosed.* *Clinical interview concluded that the patient found by touch and was self-administered the tablets of Neodicumarinum (VKA from the group of coumarins).* *The tablets were prescribed to his wife for thrombophlebitis. He had confused these tablets with the nitrates prescribed to him. The hemorrhagic coagulopathy was stopped by the administration of Prothrombin Complex Concentrate and preparation of vitamin K. The mediastinal “mass” entirely resolved. In conclusion the hemo-mediastinum was diagnosed as tumor, mis-diagnosing the hemorrhagic syndrome signs.*
*Case report 2*
*The patient K., 45, was hospitalized at the Amur Regional Clinical Hospital on 04.10.2011, due to massive sub dermal and endermic hematomas, bleeding gums and nasal bleeding.* *The patient reported that he had originally noticed skin hematomas and nasal and dermal bleedings in June, 2010. He was hospitalized at the therapeutic department of the city hospital where increased A-PPT up to 62.5 s was found (physiological range up to 35 s), a prolongation of the prothrombin time to 45.5 s and INR = 6. The coagulation was not studied in more details. The reason of coagulation failed to be determined: the patient categorically denied the administration of indirect anticoagulants; hepatic pathology, gall-bladder pathology and pathology of the intestinal tract were excluded. “DIC-syndrome of unknown etiology” was diagnosed. The patient received transfusions of fresh frozen plasma. It quickly led to the amelioration of the hemorrhagic syndrome and to the normalization of the coagulogram indexes. In 2 weeks the patient was dismissed from the hospital without signs of hemorrhagic syndrome.* *At the end of September 2011, the patient noticed again nasal bleeding. In early October the patient was admitted to the hematology department because of progressive multiple hematomas on the limbs and body. During careful questioning, the patient categorically denied the administration of any medicinal drug influencing the blood coagulation system. The patient reported diabetes mellitus type II during the last 5 years and respective treatment with Protaphane, 10 units in the morning and 10 units in the evening, with a background of suitable diet. During the examination of the liver, gall-bladder, gastro-intestinal tract and kidneys, no pathologies were found. The patient categorically denied being beaten (taking into account the location of the hematomas). The clinical blood analysis revealed anaemia (hemoglobulin – 103 g/dl) and erythrocyte sedimentation rate (ESR) of 35 mm/h. According to myelogram, hemablastosis was excluded. The coagulogram assessment revealed the following:* *➢ rapid deceleration of I-II phases of the extrinsic blood coagulation* *➢ deficiency of the factors of the prothrombin complex – prolongation of the echitoxic time (II factor), lebetoxic time (X factor) and prothrombin time (VII factor)* *➢ decreased factor IX* *➢ % V, VII, XI factors was not changed* *➢ the formation of prothrombinase through the intrinsic coagulation pathway was not affected* *➢ the final stage of coagulation was normal* *➢ moderate hyperfibrinogenemia* *➢ the activity of Antithrombin III and plasminogen was satisfactory* *➢ low level of protein C (its synthesis also depends upon Vitamin K).* *Therefore deficiency of Vitamin K-dependent factors of coagulation and of the inhibitor of coagulation protein C was diagnosed.* *The patient was further interviewed with the purpose of diagnosing the possible reasons (including criminal reasons) of the severe deficiency of K-dependent factors. To the question about rat-poison use, the patient’s answer was affirmative. From March to June 2011 and from July to September 2011, the patient, a greengrocer, without any professional help and with bare hands used rat-poison in large quantities. The active agent in rat-poison was brodifacoum. The patient reported that he had worked in a closed room, the food-store, and that he consumed himself fruit and vegetables kept in this location.* *Therefore, brodifacoum poisoning was diagnosed.* *Upon treatment start with fresh frozen plasma and 1% Vikasolum solution, 1 ml 3 times a day intravenously, the hemorrhagic syndrome recessed in 3 days. During the following month the hemorrhagic syndrome was completely resolved (with the administration of Vikasolum p.o.). Coagulogram indices normalized at the beginning of the second month.*
*Case report 3*
*A female patient, aged 60, was repeatedly admitted to different hematology units in Moscow, with severe hemorrhagic syndrome for several years. The huge bruises were noticed on the upper skin, except from areas difficult to approach by patient’s hands (the area of the vertebral spine). The round, smooth, painful lesions were palpated in the abdominal cavity (subserosal-haematomas of the intestine) and hematuria was present. All laboratory results of Vitamin K-dependent coagulopathy were present. The calm behavior of the patient, in spite of emergency of the situation as depicted by the medical personnel, made the doctor suspicious. The patient was hospitalized to the intensive care unit without clothes. When the patient’s clothes were examined, a unit package of neo-dicoumarin was discovered. The therapy with quarantine fresh frozen plasma and Vitamin K preparation led to clinical recovery and normalization of the laboratory results. During conversation with the patient, the attending physician let her know that the medical personnel had recognized the cause of her illness and she was recommended psychiatrist assistance.*
*Case report 4*
*A female patient, aged 54 years, visited doctors for several years because of bruises and hematuria.* *She was treated repeatedly in the haematology units of the city hospitals in Moscow.* *During the latest admission, huge “bruises” were observed on the mammary gland skin and on the femora skin. During the examination of her clothes, they found a package of neo-dicoumarin.* *The treatment with quarantine fresh frozen plasma and Vitamin K-preparations stopped the hemorrhagic syndrome and normalized the coagulogram. The doctor explained delicately the reason of her disease. She was recommended to stop using the dangerous medicinal drugs and to visit a psychiatrist.*
*Case report 5*
*The patient M, aged 53 years, was admitted to the hematology unit of Amur Regional Clinical Hospital from the district of Amur region with the provisional diagnosis “Hemophilia B”. He complained of “reasonless” bruises on his skin, recidivating nasal bleedings and urine discoloration (the color of the meat slops).* *He considered himself ill since June 3, 2012, when he first noticed the nasal bleeding, urine with blood mixture, in sort of meat “slops”, hematomas on his lower limbs.* *The symptomatic therapy was administered and the patient was transferred to the regional hospital. The blood test revealed a moderate post hemorrhagic anemia (erythrocytes 2.8 × 10* ^*12*^ */l, hemoglobulin 72 g/dl). Erythrocytes abundance was observed in the urine. Coagulogram revealed hypo-coagulation (after performing clotting time according to Louis White, INR, prothrombin time, APPT). A more detailed coagulogram was not done. It was not possible to determine the etiology of such coagulation. After the transfusion with fresh frozen plasma, the nasal bleeding and nephritic bleeding stopped. The results of the coagulogram tests normalized. The physicians reached a diagnosis of DIC-syndrome of undefined etiology. The patient was discharged from the hospital on 4.07.12 with improvement of his condition; the nasal bleedings and the hemorrhagic syndrome were absent.* *After 10 days the clinical picture of the hemorrhagic syndrome developed again on the skin and nasal and nephritic bleedings resumed. On 23.07.12 the patient came to the Amur Advisory Clinic. The coagulogram’s analysis revealed the increase of APPT to 60 s., PTI – 40 s., INR to 5.3 and decrease of IX blood coagulation factor (Christmas factor). He was sent to the hematology unit with the provisional diagnosis “Hemophilia B”. The elderly age of the patient, absence of hereditary history, high tolerability of the physical activity, absence of the obvious bleeding from the traumas, INR increase allowed to exclude immediately the diagnosis Hemophilia B. In the hematology unit of the Amur Regional Clinical Hospital an expanded coagulogram was firstly asked.* *The coagulogram conclusion was the following: rapid deceleration of I-II phases of extrinsic blood coagulation; factor deficiency of prothrombin-converting complex – the prolongation of the echitox time (II factor), lebetox time (X factor) and prothrombin time (VII factor), the quantity of factor IX was decreased; the percentage composition of factors V, VIII, XI was within normal limits; the formation of prothrombinase on the intrinsic coagulation pathway was not damaged; the final stage of coagulation was in the norm; the activity of antithrombin III and plasminogen was satisfactory; a low level of protein C. It was diagnosed the deficiency of Vitamin K-dependent factors of coagulation and coagulations inhibitor protein C. A light severe anemia was observed (hemoglobulin – 102 GM/DL; erythrocytes 3.2 × 10* ^*9*^ */l; leucocytes – 9.2 × 10* ^*9*^ */l, thrombocytes -250 × 10* ^*9*^ */l). According to the myelogram, hemablastosis was excluded. The biochemical analysis of the blood was within normal limits. There were erythrocytes throughout the urine. According to all these results vitamin K-dependent coagulopathy was diagnosed. The thorough questioning of the patient about possible reasons of coagulopathy including criminal motives did not lead to positive results, the patient categorically refused to admit VKA consumption and work with any rat-poison. At liver, gastro-intestinal tract and kidneys examination, pathology associated with the said coagulopathy was not revealed.* *A good clinical course was achieved with the use of PCC (Protromplex 600) and Vitamin K preparations. On the third day of the treatment, the nasal and nephritic bleedings stopped, new hematomas stopped occurring on the skin. After a month the hemorrhagic syndrome completely ended (on the background of administration of Vitamin K) and coagulogram indices normalized. The patient was discharged but physicians recorded his place of residence.* *As it was found out later, the coagulopathy had criminal origin. One of his relatives added rodenticide containing brodifacoum into the patient’s meal.*

## Therapy of VKA poisoning

Therapy for indirect coagulant poisoning includes:

1. Stopping the contact with anticoagulants.
2. Hospitalization to the intensive therapy unit without clothes for more than 24 h with the forbiddance of getting parcels from the relatives.
3. Transfusion of fresh-frozen plasma or administration of concentrated prothrombin complex. For emergency correction of anticoagulation, it’s recommended to use the three-factor prothombin complex concentration (PCC) (Prothrombinex®-HT, CSL Limited; Profilnine SD®Grifols Biologicals Inc; Uman Complex D.I. Kedrion, CastelvecchioPascoli, Italy, Prothromplex TIM 3 (Baxter, Vienna, Austria). In patients with INR 4 and in patients with higher INR levels it’s preferable to use the four-factor PCC: Prothromplex® 600, Baxter, Vienna, Austria; Confidex® CSL Behring, Marburg, Germany; Kaskadil®LFB Biomedicaments Pharmaceutical; Octaplex®Octapharma Canada; Cofact®,Sanquin Plasma Products B.V. [57].
4. Vitamin K prescription. It’s preferable to use preparations of Vitamin K_1_ and not vitamin K_3_. If the patient does not present active bleeding and there is not a necessity for the acute INR correction, it is preferable to use oral prescription of Vitamin K_1_ in a dose of 1–2 mg or in case of high INR an oral dose of 5 mg. For the treatment of the massive life threatening bleeding, Vitamin K_1_ should be injected slowly in doses of 10–20 mg, for not less than 30 min; after the intravenous administration the effect occurs in 2–4 h, the maximum effect occurs in 24 h independently of the way of administration [58–63]. The coagulogram indexes should be followed up to its complete normalization. Sometimes this takes weeks or months.

The beginning of the synthesis of blood coagulation factors after administration of Vitamin K preparations varies from 6 to 12 h and their normalization occurs in 3–5 days. That’s why the transfusion with fresh frozen plasma or administration of Prothrombin complex concentration as the source of blood coagulation factors could be more appropriate in emergency cases. Some authors point out that in severe cases of coagulopathy preparation of recombinant factor VIIa should be administered [21, 61, 64].

Therapy for warfarin intoxication should be administered for at least 15 days and for RD of the second generation or of unknown class for at least 1 month. The decision for therapy termination is complex and is based on the time period past from drug discontinuation and the results of blood coagulation tests in the first 36–48 or 96 h after therapy discontinuation.

## Conclusion

Main reasons of acquired Vitamin K-dependent coagulopathies are: the hidden administration of anticoagulants of indirect action from patients with hysteria and other psychiatric disorders; the accidental use of VKA instead of other drugs; consumption of significant doses of VKA or rat-poison with meals in cases of suicide or foul play; accidental contact with substances containing VKA or rat-poison; disregard of the working rules with rat-poisons containing VKA. If there is no memory of indirect anticoagulants use, the differential diagnosis is difficult. Differential diagnosis of Vitamin K-deficiency is necessary in cases of intestinal disbacteriosis; enteropathies accompanied by heavy diarrhea; mechanical jaundice with acholia that leads to reduction or absence of bile secretion into the intestinal tract and mal-absorption of Vitamin K; prematurity of the newborns that is associated with subnormal synthesis of vitamin K in the intestinal tract or severe liver disease (acute dystrophies, hepatitis, cirrhosis). In cases where the coagulogram is typical of Vitamin K-deficiency, even if its etiology is not determined, immediate prescription of the appropriate therapy is needed: administration of fresh-frozen plasma or Prothrombin Complex Concentrate (the last one is preferable), prescription of Vitamin K preparations and symptomatic therapy. With early diagnosis and prescription of adequate therapy, prognosis is favorable.

## Abbreviations

APTT: Activated partial thromboplastin time’s
AS: Active substance
DIC: Disseminated intravascular coagulation syndrome
INR: International normalized ratio
MSBP: Munchausen syndrome by Proxy
PCC: Prothombin complex concentration
RDs: Rodenticides
Vitamin K_3_: 2-methyl-1,4-naphthoquinone
Vitamin K_4_: 2-methyl-1,4-naphtho-hydroquinone
Vitamin K_5_: 2-methyl-4-amino-1-naphtho-hydroquinone
Vitamin K_6_: 2-methyl-1,4-naphthoquinone diamine
Vitamin K_7_: 3-methyl-4-amino-1-naphtho-hydroquinone
VKA: Vitamin K antagonists
